# Breaking Healthcare Barriers for Transgender Individuals With Rare Tumor Presentation

**DOI:** 10.7759/cureus.33791

**Published:** 2023-01-15

**Authors:** Christopher J Vaccaro, Margaret Grace McGrath, Erin McFadden

**Affiliations:** 1 College of Medicine, A.T. Still University School of Osteopathic Medicine in Arizona, Mesa, USA; 2 Internal Medicine, A.T. Still University School of Osteopathic Medicine in Arizona, Mesa, USA

**Keywords:** cancer, cultural competency, testicular cancer, germ cell tumor, transgender, case report

## Abstract

Transgender persons can experience healthcare barriers and potentially suffer from preventable health disparities. Some challenges these individuals may face include the lack of provider education, social stigma, socioeconomic barriers to care, and insurance instability. Combating this problem requires systemic changes. Unfortunately, there are limited data on providers’ perspective on taking care of transgender persons, and healthcare delivery systems are often unequipped to adequately manage these patients. This case presentation exemplifies many of these challenges.

A 47-year-old transgender female with a history of testicular cancer, presented with bleeding from a lump on her neck. A computed tomography (CT) scan of the neck revealed a large mass suspicious of malignancy. Pathology identified metastatic colorectal adenocarcinoma. Esophagogastroduodenoscopy, colonoscopy, positron emission tomography scan, CT abdomen/pelvis, and serum tumor marker showed no evidence of a primary gastrointestinal malignancy.

This presentation likely represents a late relapse of a residual, metastatic germ cell tumor with malignant somatic transformation. This case was greatly impacted by social determinants of health. The patient did not identify with her male anatomy, which delayed the detection of the initial testicular malignancy. In the post-operative period, the patient did not attend follow-up appointments to avoid discussing her male genitalia. When tumor relapse did occur, the patient experienced financial, insurance, and transportation instability; this delayed medical care and allowed the mass to grow to an extraordinary size.

## Introduction

According to the Williams Institute, there are approximately 1.3 million transgender individuals, which comprise 0.5% of the United States adult population [1]. Additionally, the number of people identifying as transgender has increased with each successive generation; 2.1% of Generation Z, people born between 1993 and 2005, identify as transgender [2]. With a growing population of people who identify as transgender, it is imperative for medical providers to provide individualized and culturally sensitive care appropriate for their physical anatomy. 

Transwomen have the potential to experience greater gender dysphoria and dissociation with their male genitalia. According to Gil-Llario et al., “trans women had a much more ego-dystonic and problematic experience of their sexuality than trans men” [3]. Additionally, transgender women experienced greater rejection of their genitals and did not want their genitals to be touched. Healthcare providers should understand that transwomen must persevere through more adversity to feel safe and accepted in society. 

According to Mirza and Rooney, 23% of transgender patients said a provider “intentionally misgendered them or used the wrong name” [4]. Transgender persons are more likely to be affected by “…subtle, apparently insignificant features of healthcare spaces and interpersonal interactions called microaggressions” [5]. Oftentimes, unintentional microaggressions can add to the stress of the patient, exacerbating the problem at hand. Furthermore, fear of discrimination and mistreatment due to gender identity can deter transgender patients from seeking care. 

In addition to socioeconomic and societal barriers faced by transgender persons, physicians also experience unique challenges in caring for these patients. It is the physician's responsibility to create a safe space for patients to identify with their true selves. This includes respecting and celebrating patients’ gender identity. However, physicians are also responsible for taking care of a patient's physical anatomy even when it is incongruent with their gender identity. This case highlights these challenges and is an excellent example of the barriers that both transgender patients and their physicians face. 

Testicular carcinoma is the most common cancer affecting young biological males. An estimated 9,470 new cases were diagnosed in 2021. One out of every 270 biological males will be diagnosed with testicular cancer in their lifetime. Fortunately, the five-year survival rate is 94.9% [6]. The incidence of testicular cancer in transwomen undergoing hormone therapy is the same as that in biological men [7]. Testicular cancer behavior is very complex and is dependent on the age of onset, histology, and clinical stage at presentation. Germ cell tumors (GCTs) are responsible for 95% of testicular neoplasms and originate from primordial germ cells [8]. GCTs consist of embryonal carcinomas, yolk sac tumors, choriocarcinomas, and teratomas. Testicular GCTs primarily spread via the lymphatic system and frequently present with metastatic lesions. Baniel et al. conducted the largest study with 81 patients, noting 56% of patients presented with metastases and 34% having distant metastatic lesions [9]. It is worth noting that in patients with a large tumor burden at initial presentation malignant cells are more likely to migrate to the supraclavicular lymph nodes [10]. 

GCTs have stem-cell-like properties in various somatic tissues such as teeth, hair, bone, etc. Malignant somatic transformation (MST) occurs in 3%-6% of GCTs [11]. Sarcomas represent the most commonly observed transformed histology, along with primitive neuroendocrine tumors, carcinomas, and adenocarcinomas. GCTs with MST are highly chemotherapy-resistant and not widely amenable to chemotherapy or radiation. The primary prognostic factor to prevent disease recurrence is complete surgical resection [12]. Incomplete margins greatly increase relapse possibility.

Most relapses occur within two years, with the most common locations being the retroperitoneum, lung, and mediastinum [13]. A late relapse of a GCT is defined as previously diagnosed GCT, with subsequent recurrence of GCTs, more than two years following successful treatment of previous GCTs [13]. Late relapses occur in 1.4% of seminomas and 3.2% of non-seminomatous GCTs [14]. Late relapse can occur following a complete response to initial testicular GCT treatment, with 36% of late relapses occurring 10 years later [13]. The resulting tumor from a late relapse is more likely to contain malignant somatic mutation [15]. Additionally, adenocarcinoma is associated with a delayed relapse interval time, occurring decades after complete response to treatment [15-17].

## Case presentation

A 47-year-old Caucasian transgender female presented to the Emergency Department with a chief complaint of “bleeding from a lump on neck”. The neck mass was first noticed four years ago; however, she noticed a rapid size increase over the past six months, which was associated with fatigue and a 30-pound unintentional weight loss. She presented to the Emergency Department that day due to the mass ulcerating through the skin, producing some serosanguinous drainage. The patient denied hoarseness, dysphonia, dysphagia, night sweats, fever, and chills. The patient delayed medical care due to financial insecurity and insurance issues. 

Past medical history was relevant for testicular carcinoma of unknown histology. It was treated 20 years ago with right orchiectomy and an unknown adjuvant chemotherapy regimen. The patient also suffered from bipolar disorder and was not taking any medications. She had a family history significant for cancer. 

Her mother had lung cancer, while her father and paternal grandfather had colon cancer. She denied undergoing any gender-affirming surgery or hormonal therapy. The patient refused to discuss the time in her life when she identified as a male. She currently lives alone and collects disability due to her bipolar disorder. The patient admitted to a seven-pack-year smoking history and successfully quit 20 years ago. A review of systems pertinent to fevers and weight loss was done. The patient denied any neurological, cardiovascular, or gastrointestinal symptoms. The patient declined to answer questions regarding her genitourinary tract. 

**Figure 1:**
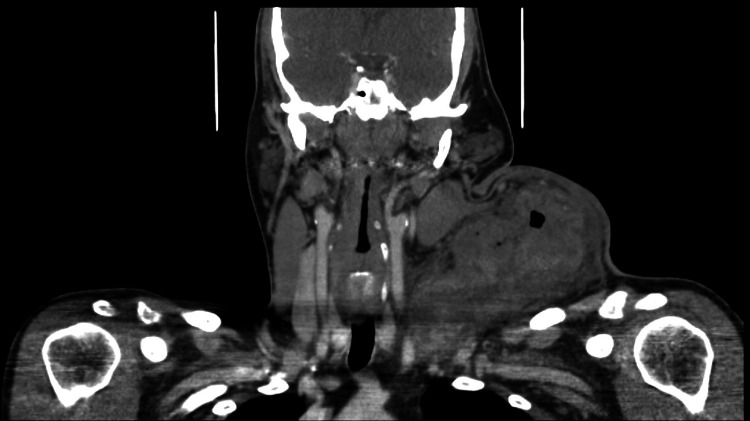
Coronal image of soft tissue neck obtained using CT scan with contrast revealed that there was a large, complex, ulcerated mass in the left neck measuring approximately 12 x 10 x 15 cm. The median margin abuts the carotid sheath and there is attenuation of the internal jugular vein. The inferior margin approaches the thoracic inlet including the great vessels. This has the appearance of a necrotic, ulcerated malignancy with associated inflammation. CT: Computed tomography

**Figure 2:**
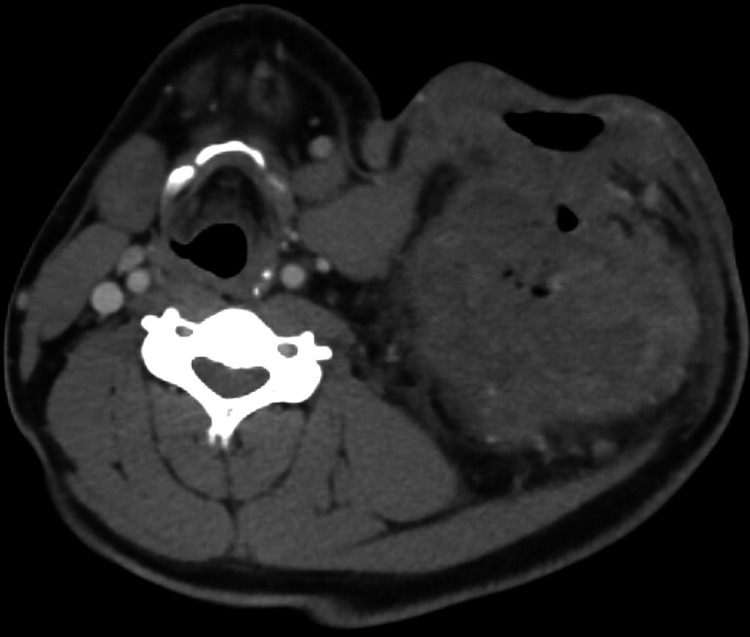
Cross-sectional image of soft tissue neck obtained using CT scan with contrast revealed that there was a large, complex, ulcerated mass in the left neck measuring approximately 12 x 10 x 15 cm. The median margin abuts the carotid sheath and there is attenuation of the internal jugular vein. The inferior margin approaches the thoracic inlet including the great vessels. This has the appearance of a necrotic, ulcerated malignancy with associated inflammation. CT: Computed tomography

Physical exam revealed a 15 cm mass located on the left lateral aspect of the left neck base, with an opening of 2 cm and minimal bleeding, surrounded by an area of ulceration. The remainder of the exam was grossly normal other than stated above. Genitourinary exam was deferred. CT scan of the neck was ordered to further evaluate the mass, and the CT images are displayed in Figure 1 and Figure 2.

CT scan of the chest was unremarkable and confirmed no involvement of great vessels. Aside from a gram-negative UTI, labs are within normal limits. 

Fine needle aspiration biopsies were obtained and were inconclusive necessitating an incisional biopsy. Results showed fibro-skeletal tissue exhibiting keratin deposition and cholesterol clefts with giant cell reaction, chronic lymphohistiocytic inflammation, and microcalcifications. Findings were suggestive of a ruptured keratinous-type cyst with no evidence of malignancy.

Due to the massive size, proximity to the great vessels, and cosmetic appearance, the patient opted for total tumor removal via left radical neck dissection. The mass appeared to be well encapsulated with significant fibrosis. It extended into the posterior cervical triangle and down into the superior mediastinum. The mass was pedicled onto the left subclavian vein and resected in its entirety. 

The neck mass specimen was sent for permanent pathology which reported that the tumor showed columnar cells in glandular formation associated with extensive necrosis. Immunostains showed positive AE1/3, CAM 5.2, CK 20, CDX2, villin, and glypican-3; focally positive for EMA; weakly positive for SALL4; and negative for CK7, AFP, inhibin, OCT 3/4, PLAP, CD 30, GATA-3, PAX 8, RCC, thyroglobulin, and TTF-1. The proliferation index ki-67 is 90%. The findings are consistent with a metastatic colorectal adenocarcinoma and exclude testicular yolk sac, embryonal, seminoma and urothelial, breast, renal cell and thyroid carcinoma. However, given the patient’s history of testicular carcinoma, a metastatic colorectal-type adenocarcinoma from a teratomatous component of the testicular carcinoma cannot be entirely excluded and is highly probable. Four lymph nodes were sampled, which were negative for malignancy. 

Gastroenterology was consulted to identify the primary source and ordered a comprehensive workup. CT abdomen/pelvis w/ contrast was concerning for a possible malignant lymph node within the retroperitoneum. Colonoscopy and esophagogastroduodenoscopy revealed no evidence of malignancy. CEA, PSA, and CA 19-9 were within normal limits. Positron emission tomography (PET) scan revealed no hypermetabolic activity, ruling out a possible malignant retroperitoneal lymph node.

Without locating a primary gastrointestinal malignancy, oncology was consulted for further evaluation. Oncology reviewed the case and had follow-up imaging. They determined that no chemotherapy was indicated. PET scans have been repeated every six months for the past four years with no evidence of malignancy. 

## Discussion

Testicular cancer is the most common cancer in males 15-30 years old and transgender females have the same risk for this cancer as cis-males. Testicular malignancies are either of germ cell origin, sex cord-stromal tumors or mixed with various cell lines. Testicular GCTs are malignancies of pluripotent cells that retain the ability to differentiate into cell lines of any embryonic layer. Rarely these tumors can undergo MST by way of a mechanism that is poorly understood. Additionally, GCTs with MST can lie dormant for years. 

GCTs have known pluripotent cell differentiation potential, but the mechanism of MST is poorly understood. Umbreit et al. in 2020 performed genetic analysis on testicular GCTs in an attempt to understand the mechanism behind MST [18]. They hypothesized that MST arises from a common clonal origin via the phenomenon of microchimerism. Progenitor stem-like cells are subject to epigenetic changes, and through the process of microchimerism, cells can migrate to distant sites, differentiate into various cellular lineages, and remain dormant for a prolonged period [18]. The microcellular environment within the supraclavicular lymph node likely induced the expression of certain epigenetic phenotypes inducing the transformation and growth of colorectal adenocarcinoma.

The lymphatic system, filled with naïve lymphocytes, can act as an immune shelter for a GCT with MST. GCTs with MST under epigenetic pressures can express stem-like or somatic antigen evading immune recognition. A small chemotherapy-resistant malignant cell population can lie dormant, variably expressing self-antigen, possibly accumulating genetic mutations, until a positive driving mutation triggers rapid cell division. The time period to relapse is highly variable; however, adenocarcinoma is the histology most associated with a delayed relapse interval [15-17].

Testicular GCT relapse is a known complication necessitating the need for extended outpatient follow-up. Dieckmann et al. found that 61% of asymptomatic relapses can be discovered with routine follow-up [19]. Late relapse most commonly manifests as lymphadenopathy within the chest, retroperitoneum, or neck. Recurrence can be assessed with serum AFP and hCG; however, these markers have been shown to be unreliable. Periodic chest x-rays and CT scans are more reliable indicators of disease but require additional radiation exposure to patients with a history of a tumor that is highly sensitive to transformation. Relapse surveillance is dependent upon patient compliance with appointments and sensitive physical examinations. 

This patient identified as a female at the age of 13 in an era when it was extremely taboo to identify as the opposite gender. She did not recognize her male genitalia as a part of her body. Thus, when a testicular mass eventually developed at the age of 20, she did not feel comfortable speaking to a medical provider regarding anatomy she did not feel connected to. The effect of this delay allowed for a large testicular tumor burden to develop, send off micrometastases to the neck, and then undergo MST. Once the MST had occurred, subsequent chemotherapy was ineffective. Gender dysphoria and the cognitive dissonance this patient experienced with her birth anatomy drove the disease process further. 

At the time of relapse, she experienced insurance difficulties, financial insecurity, and transportation issues further complicating prompt treatment of the neck mass. In the United States, transgender adults are nearly twice as likely to be uninsured and generally have lower incomes as compared to cisgender adults [20]. Reducing these barriers of access to equitable care is paramount in providing timely and effective treatment for transgender patients. In this case, the patient’s financial insecurity and lack of health insurance delayed additional care and allowed the tumor to reach an extraordinary size. This patient denied certain lab tests and imaging studies for fear of the insurance company denying her claims, saddling her with financial debt in a time of turmoil. When working with any patient, particularly one with a case as complex as this, the socioeconomic determinants of health must be factored into the decision-making of the physician. Fear of the financial burden associated with the medical therapy for the mass on her neck limited the types of interventions that could be done to treat the patient’s condition. 

This patient most likely developed colorectal adenocarcinoma of the neck due to metastatic cells from a GCT that underwent MST. We encourage physicians to keep a broad differential diagnosis in the workup of malignancy for recurrence of a testicular GCT, regardless of the remission period. The diagnosis of a testicular malignancy and its sequelae can be especially harmful for transgender women.

## Conclusions

Physicians play a crucial role in establishing a safe and nurturing environment for patients. However, physicians often lack adequate training in how to care for transgender patients. Moving forward as physicians, we need to embrace patients however they identify themselves and provide a comfortable environment to assess and screen their past and present anatomy. Physicians need to be cognizant of their patients' preferred pronouns, refrain from microaggressions, and remain constantly aware of their implicit biases. Providing culturally competent patient-centered care provides a space for transgender patients to express their gender identity, while simultaneously acknowledging their physical anatomy. 

One way to ensure that healthcare systems capture both patients’ gender identity and physical anatomy is to create an “Anatomy Inventory” for every new patient. Upon establishing care, all patients will be asked to fill out an “Anatomy Inventory” prior to being seen by the physician. This form would include the patients’ biological sex, birth anatomy, sexual orientation, gender identity, any gender affirmation surgery the patient has had done, and current medications including hormones. It is important that this form is updated on a yearly basis so that the physician can be able to provide culturally competent care, while also addressing preventative care specific to the patient’s physical anatomy. An “Anatomy Inventory” provides an opportunity for both the physician and the patient to start a healthy conversation about the patient’s physical health and their unique healthcare needs. This case could have been prevented had the patient felt comfortable in her own body. The dissociation of the patient with her genitals likely caused the delay in care and allowed the tumor to undergo MST to a chemo-resistant mass. Additionally, once the mass recurred on her neck decades later, she experienced systemic, societal, and socioeconomic barriers.
